# Pharmacological Actions of Myricetin in the Nervous System: A Comprehensive Review of Preclinical Studies in Animals and Cell Models

**DOI:** 10.3389/fphar.2021.797298

**Published:** 2021-12-16

**Authors:** Jie Li, Haitao Xiang, Chao Huang, Jiashu Lu

**Affiliations:** ^1^ Department of Gastroenterology, The People’s Hospital of Taizhou, The Fifth Affiliated Hospital of Nantong University, Taizhou, China; ^2^ Department of Neurosurgery, Suzhou Kowloon Hospital, Shanghai Jiaotong University School of Medicine, Suzhou, China; ^3^ Department of Pharmacology, School of Pharmacy, Nantong University, Nantong, China; ^4^ Department of Pharmacy, The People’s Hospital of Taizhou, The Fifth Affiliated Hospital of Nantong University, Taizho, China

**Keywords:** myricetin, neuroinflammation, oxidative stress, brain, flavonoid

## Abstract

Myricetin is a natural flavonoid extracted from a variety of plants, such as medicinal herbs, vegetables, berries, and tea leaves. A growing body of evidence has reported that myricetin supplementation display therapeutic activities in a lot of nervous system disorders, such as cerebral ischemia, Alzheimer’s disease, Parkinson’s disease, epilepsy, and glioblastoma. Myricetin supplementation can also protect against pathological changes and behavioral impairment induced by multiple sclerosis and chronic stress. On the basis of these pharmacological actions, myricetin could be developed as a potential drug for the prevention and/or treatment of nervous system disorders. Mechanistic studies have shown that inhibition of oxidative stress, cellular apoptosis, and neuroinflammatory response are common mechanisms for the neuroprotective actions of myricetin. Other mechanisms, including the activation of the nuclear factor E2-related factor 2 (*Nrf2*), extracellular signal-regulated kinase 1/2 (ERK1/2), protein kinase B (Akt), cyclic adenosine monophosphate-response element binding protein (CREB), and brain-derived neurotrophic factor (BDNF) signaling, inhibition of intracellular Ca^2+^ increase, inhibition of c-Jun N-terminal kinase (JNK)-p38 activation, and suppression of mutant protein aggregation, may also mediate the neuroprotective effects of myricetin. Furthermore, myricetin treatment has been shown to promote the activation of the inhibitory neurons in the hypothalamic paraventricular nucleus, which subsequently produces anti-epilepsy effects. In this review, we make a comprehensive understanding about the pharmacological effects of myricetin in the nervous system, aiming to push the development of myricetin as a novel drug for the treatment of nervous system disorders.

## Introduction

**FIGURE 1:**
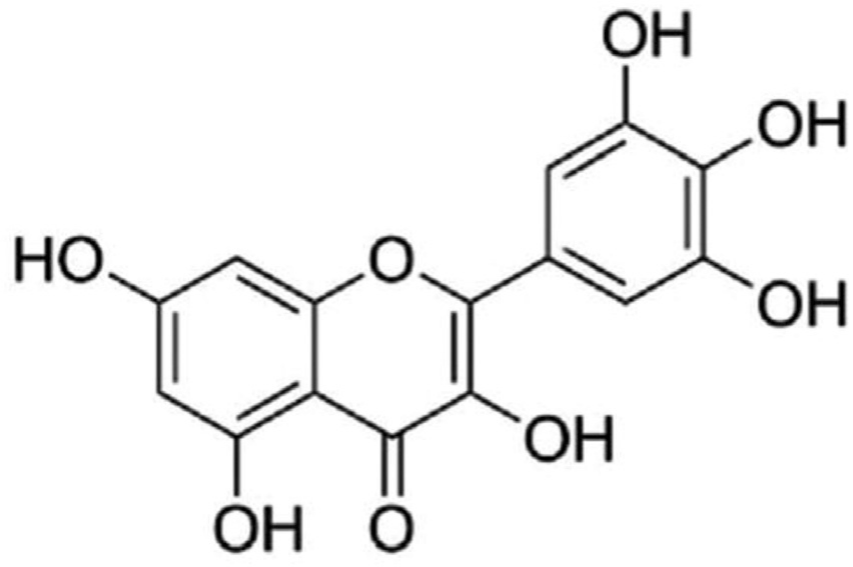
The structure of myricetin.

Myricetin (Figure 1) is a natural flavonoid extracted from a variety of plants, such as vegetables, berries, and tea leaves (Gupta et al., 2020). It can produce numerous pharmacological effects, including anti-inflammation (Jang et al., 2020), anti-oxidation (Wu et al., 2016), anti-carcinogen (Xie et al., 2020), anti-diabetes (Lalitha et al., 2020), and cardio-protection (Qiu et al., 2017), and display therapeutic activities in central nervous system disorders like cerebral ischemia (Wu et al., 2016; Sun et al., 2018), Parkinson’s disease (Dhanraj et al., 2018), Alzheimer’s disease (Liu et al., 2020), depression (Ma et al., 2015), epilepsy (Sun et al., 2019), and glioma (Li et al., 2021). Pharmacodynamic studies have revealed that myricetin has a series of deficits. Firstly, its oral bioavailability is only about 9.62 and 9.74%, respectively, at 2 oral doses 50 and 100 mg/kg, indicating a poor absorbing property (Dang et al., 2014). Secondly, the stability of myricetin is easily influenced by gastrointestinal environment, which is stable in simulated gastric fluids and buffer solutions with low pH values and experience a pseudo-first-order kinetic degradation in simulated intestinal fluids and buffer solutions with high values (Xiang et al., 2017). These deficits indicate that orally administration of myricetin can induce a state with low efficacy in brain delivery which may limit the application of myricetin in clinical practice. However, this hypothesis may be not definitely true, as the neuroprotective effects of myricetin produced by orally administration have been observed repeatedly in previous studies. For example, myricetin administration has been shown to reduce infarct brain volume in rat models of cerebral ischemia (Wu et al., 2016; Sun et al., 2018). Myricetin administration can also improve learning and memory impairment in animals who receive streptozotocin (Ramezani et al., 2016), D-galactose (Lei et al., 2012), or scopolamine (Wang et al., 2017) stimulation. In addition, myricetin administration can ameliorate the pathogenesis of Parkinson’s disease in models *in vitro* and *in vivo* (Dhanraj et al., 2018; Deng et al., 2021). The discrepancy of myricetin between its low oral bioavailability and neuroprotective effects could be coordinated by a metabolism hypothesis: the orally administered myricetin could be converted into a metabolite that penetrates the blood-brain-barrier. Mechanistic studies have revealed that inhibition of neuroinflammation, oxidative stress, and cellular apoptosis (Jang et al., 2020; Boriero et al., 2021; Deng et al., 2021; Hamdi et al., 2021; Pluta et al., 2021), activation of nuclear factor E2-related factor 2 (*Nrf2*) (Wu et al., 2016), extracellular signal-regulated kinase 1/2 (ERK1/2) (Lei et al., 2012; Jang et al., 2020), protein kinase B (Akt) (Sun et al., 2018), and cyclic adenosine monophosphate-response element binding protein (CREB) (Lei et al., 2012), inhibition of intracellular Ca^2+^ increase (Oyama et al., 1994; Chang et al., 2015), and inhibition of c-Jun N-terminal kinase (JNK) (Jang et al., 2020) or p38 (Hagenacker et al., 2010; Sun et al., 2018; Jang et al., 2020) may mediate the neuroprotective actions of myricetin. On the basis of these reports, we conclude that myricetin, dependent on its metabolites which possess neuroprotective activities, could be developed as a potential candidate for the treatment of nervous system disorders. In this review, we make a comprehensive outline for the pharmacological actions and possible mechanisms of myricetin in the nervous system by searching online literatures and databases involving *in vivo* animal studies and *in vitro* cell experiments, aiming to push the development and application of myricetin in nervous system disorder treatment.

## Pharmacological Effects of Myricetin in Cerebral Ischemia

**FIGURE 2:**
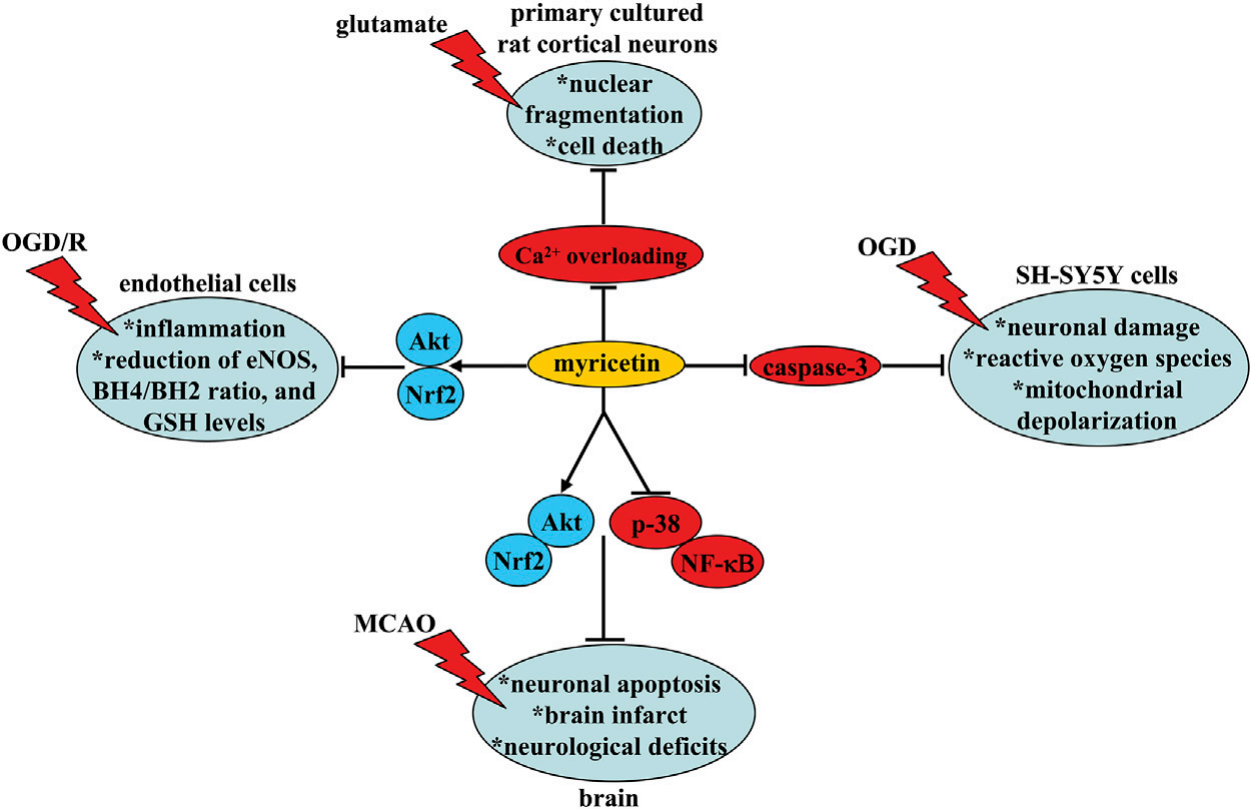
Neuroprotective effects and possible mechanisms of myricetin in models of cerebral ischemia. Myricetin inhibits (i) OGD/R-induced inflammation, decrease in eNOS expression, phosphorylation, and activity, decrease in intracellular BH4/BH2 ratio, and decrease in intracellular GSH levels in endothelial cells via stimulation of Akt and Nrf2, (ii) glutamate-induced nuclear fragmentation and cell death in primary cultured rat cortical neurons via suppression of Ca^2+^ overloading, and (iii) OGD-induced neuronal damage, reactive oxygen species production, and mitochondrial depolarization in SH-SY5Y cells via inhibition of caspase-3. Myricetin administration can also reduce neuronal apoptosis and infarct area and improve neurological deficits in a rat model of MCAO via increasing Akt activity, decreasing p38 and NF-κB activity, and increasing Nrf2 nuclear translocation.

Cerebral ischemia is a common cerebral vascular disease which occurs in conditions like stroke (Wang et al., 2021) and hypo-perfusion (Ciacciarelli et al., 2020). Oxido-nitrosative stress and neuroinflammation are two key pathological events that can mediate the pathogenesis of cerebral ischemia (Chen et al., 2020; Takeda et al., 2021). Inhibition of oxido-nitrosative stress and neuroinflammation may be beneficial for the treatment of cerebral ischemia. Orally myricetin administration once daily at the dose of 5 and 25 mg/kg (7 days) (Sun et al., 2018) or at the dose of 10 or 20 mg/kg [2 h before and every day after middle cerebral artery occlusion (MCAO)] (Wu et al., 2016) has been shown to reduce neuronal apoptosis and infarct area and improve neurological deficits in a rat model of permanent MCAO *via* suppressing abnormally increased pro-inflammatory cytokines, malondialdehyde (MDA), and reactive oxygen species, and abnormally decreased GSH, superoxide dismutase (SOD) activity, mitochondrial membrane potential, and adenosine triphosphate (ATP) levels in ischemic brain tissues (Sun et al., 2018; Wu et al., 2016) (Figure 2; Table 1). These effects of myricetin may be associated with the increase in Akt activity and Nrf2 nuclear translocation and the decrease in p38 or nuclear factor-κB (NF-κB) activity (Wu et al., 2016; Sun et al., 2018) (Figure 2; Table 1). *S*tudies *in vitro* have shown that myricetin pre-incubation (10 nM, 3 h) can attenuate oxygen-glucose deprivation (OGD)-induced neuronal damage, reactive oxygen species production, and mitochondrial depolarization in SH-SY5Y cells, with a possible mechanism involving a direct binding and inhibition of myricetin to caspase-3 (Wu et al., 2016; Figure 2 and Table 1). This indicates that reducing oxidative stress may contribute to the neuroprotective effect of myricetin in cerebral ischemia.

**TABLE 1:** Comprehensive information about the effects of myricetin in *in vivo* and *in vitro* models cerebral ischemia.

Pharmacological effect	Object	Drug administration	Possible mechanisms	References
*Reduce neuronal apoptosis	Rat	*5, 25 mg/kg	*Suppress pro-inflammatory cytokine, MDA, and reactive oxygen species production	Sun et al. (2018)
*Reduce infarct area *improve neurological deficits		*i.g	*Increase GSH production and SOD and Akt activity	
—		*Once daily, 7 days	*Decrease NF-κB activity	
*Reduce neuronal loss and apoptosis	Rat	*10, 20 mg/kg	*Reduce MDA and reactive oxygen species production	Wu et al. (2016)
*Reduce infarct area		*i.g	*Increase Nrf2 function	
*Improve neurological deficits		*2 h before and every day after MCAO	*Improve mitochondrial function	
*Improve learning and memory function		—	—	
Attenuate OGD-induced neuronal damage, reactive oxygen species production, and mitochondrial depolarization	SH-SY5Y cells	*10 nM	*Inhibit caspase-3 activity	Wu et al. (2016)
		*3 h before ODG exposure		
Prevent glutamate-induced nuclear fragmentation and cell death	Rat cortical neurons	*0.1, 0.3, 1, 3, 10 μM	Suppress Ca^2+^ overloading, reactive oxygen species production, and caspase-3 activation	Shimmyo et al., 2008
		*24 h of pretreatment along with 24 h of simultaneous treatment		
Reduce 4-AP-induced Ca^2+^ influx, neuronal depolarization, and glutamate release	Isolated nerve terminals	30 μM co-incubation	Block the N-type and P/Q-type Ca^2+^ channel	Chang et al. (2015)
*Reduce OGD/R-induced pro-inflammatory cytokine production and decrease in eNOS expression/phosphorylation/activity, BH_4_/BH_2_ ratio, and GSH levels	HBMECs	*10, 30, 60 μM	Activate the Akt and Nrf2 signal	Zhang et al. (2019)
		*Pretreatment, 24 h		

The Ca^2+^ overloading-mediated neuronal excitotoxicity is a well-known mechanism for the pathogenesis of cerebral ischemia, during which the abnormal increase in intracellular Ca^2+^ contributes largely to neuronal loss (Kobayashi and Mori, 1998). Although no direct evidence can support the down-regulatory effect of myricetin on intracellular Ca^2+^ in the anti-cerebral ischemia effect of myricetin, some *in vitro* studies may provide indirect evidence. For example, myricetin pretreatment (0.1, 0.3, 1, 3, 10 μM, 24 h) has been shown to prevent glutamate-induced nuclear fragmentation and cell death in primary cultured rat cortical neurons by suppressing the N-methyl-D-aspartic acid (NMDA) receptor-mediated Ca^2+^ overloading, reactive oxygen species production, and caspase-3 activation (Shimmyo et al., 2008; Figure 2 and Table 1). Myricetin incubation (30 μM) can also reduce Ca^2+^ influx, neuronal depolarization, and glutamate release in isolated nerve terminals induced by a potassium channel blocker 4-aminopyridine (4-AP) via blocking the N-type and P/Q-type Ca^2+^ channels (Chang et al., 2015; Figure 2 and Table 1). However, whether inhibition of Ca^2+^ overloading indeed contributes to the neuroprotective actions of myricetin should be ascertained in future studies.

The dysfunction of the endothelium is a known pathological event that mediates the impairment in blood-brain-barrier in cerebral ischemia (Andjelkovic et al., 2019). Methods that restore the function of the endothelium may help to ameliorate neuronal damages in cerebral ischemia. Myricetin pre-incubation (10, 30, or 60 μM, 24 h) can ameliorate OGD/R-induced production of pro-inflammatory cytokines, decrease in endothelial nitric oxide synthase (eNOS) expression/phosphorylation/activity, decrease in intracellular tetrahydrobiopterin (BH_4_)/BH_2_ ratio, and decrease in intracellular glutathione (GSH) levels in human brain micro-vessel endothelial cell (HBMECs) (Zhang et al., 2019; Figure 2 and Table 1), which may indicate a state of eNOS uncoupling and permeability increase. These effects of myricetin may be dependent on Akt or Nrf2, as inhibition of Akt or Nrf2, both of which could be activated by myricetin, can block the improvement effect of myricetin on eNOS activity and endothelial permeability (Zhang et al., 2019; Figure 2 and Table 1). These results uncover a possibility that improving the functions of the endothelium may contribute to the anti-cerebral ischemia effect of myricetin. In view of the importance of the endothelium in brain homeostasis (van Ierssel et al., 2014), researchers should further establish the causal relationship between the regulatory effect of myricetin on cerebral ischemia and endothelial cells in future studies.

## Pharmacological Effects of Myricetin in Alzheimer’s Disease

**FIGURE 3:**
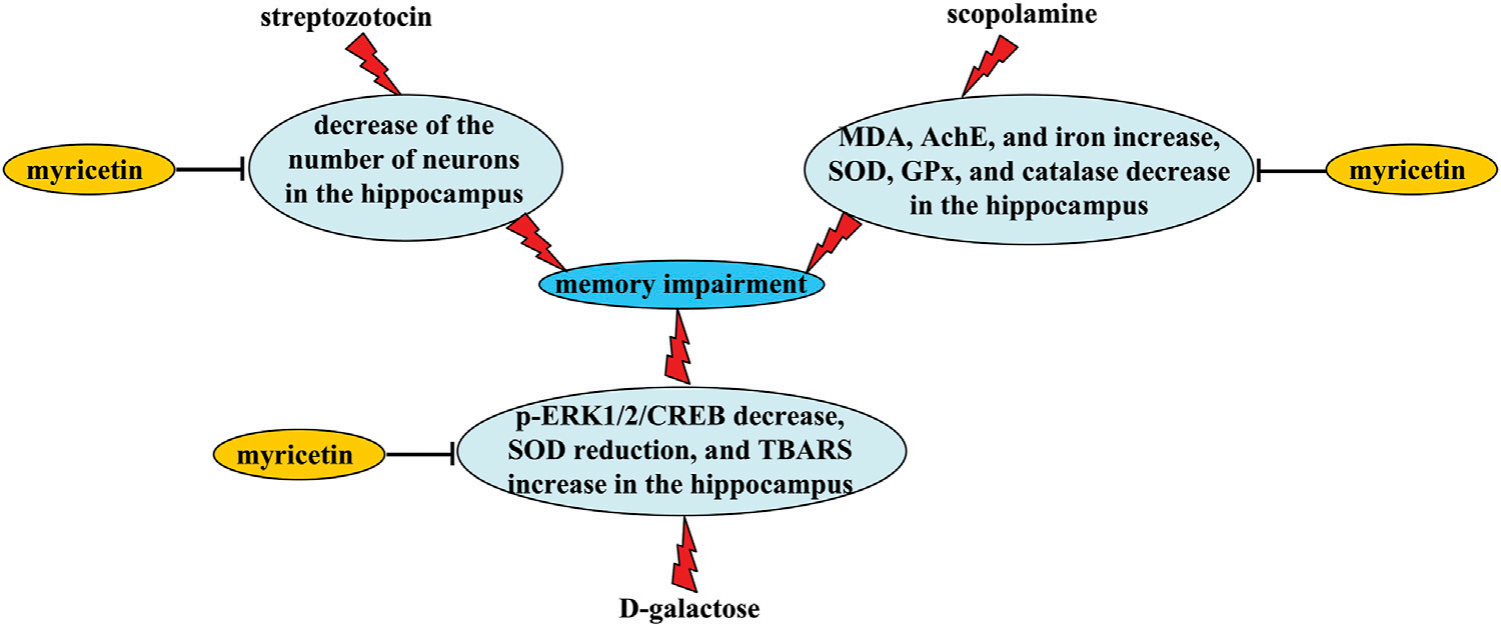
Neuroprotective effects and possible mechanisms of myricetin in Alzheimer’s disease-associated models. Myricetin administration can suppress (i) streptozotocin-induced memory impairment via inhibiting streptozotocin-induced decrease in neuronal numbers in the hippocampus, (ii) D-galactose-induced memory impairment via inhibiting scopolamine-induced increase in MDA levels and iron contents and decrease in SOD, GPx, and catalase activities in the hippocampus, and (iii) D-galactose-induced memory impairment via inhibiting D-galactose-induced decrease in the phospho-ERK1/2 and -CREB, decrease in SOD activity, and increase in TBARS levels in the hippocampus.

Alzheimer’s disease (AD) is a common neurodegenerative disease with progressive worsening in cognition- and memory-associated neuronal functions (Rahimi, 2021). To date, no definite drugs could be available for the treatment of AD, which is partially due to the deficiency of the understanding about the pathological mechanisms of AD. Therefore, it is urgent to search novel drugs for the treatment of AD. Myricetin could be a drug for that purpose. Firstly, myricetin administration (5 or 10 mg/kg, i. p., started 1 day before stereotactic surgery, 21 days) can suppress memory impairment in a rat model of AD induced by streptozotocin (Ramezani et al., 2016; Figure 3 and Table 2). Secondly, orally myricetin administration (100 mg/kg, once daily, 8 weeks) has been shown to suppress D-galactose-induced memory impairment in mice, which was indicated by the decrease in the number of platform crossings, the decrease in the time and distance spent in the target quadrant, and the increase in the time of first crossing (Lei et al., 2012; Figure 3 and Table 2). Thirdly, orally myricetin administration (25 or 50 mg/kg, once daily, 6 days) can suppress scopolamine-induced decreases in platform crossings and swimming time that spent in the target quadrant in the Morris Water Maze (*MWM*) test in mice (Wang et al., 2017; Figure 3 and Table 2).

**TABLE 2:** Comprehensive information about the effects of myricetin in animal models Alzheimer’s disease.

Pharmacological effect	Object	Drug administration	Possible mechanisms	References
Suppress streptozotocin-induced memory impairment	Rat	*5, 10 mg/kg	*Increase neuronal numbers in the hippocampus	Ramezani et al. (2016)
		*i.p		
		*1 day before stereotactic surgery, 21 days		
Suppress D-galactose-induced memory impairment	Mouse	*100 mg/kg	*Up-regulate *p*-ERK1/2/CREB	Lei et al. (2012)
		*i.g	*Increase SOD activity	
		*once daily, 8 weeks	*Decrease TBARS levels	
Suppress scopolamine-induced decrease in platform crossings and swimming time spent in the target quadrant in the Morris Water Maze test	Mouse	*25, 50 mg/kg	*Reduce *MDA* levels and AChE activity, and increase SOD, GPx, catalase activity in the hippocampus	Wang et al. (2017)
		*i.g		
		*once daily, 6 days		
Suppress Aβ_1-42_-induced nuclear fragmentation and caspase-3 activation	Cortical neurons	*0.3, 1, 3, 10 μM	*Promote ADAM10 expression	Shimmyo et al., 2008
Reduce Aβ_1-40_/Aβ_1-42_ levels		*Pretreatment 24 h along with 24 h of simultaneous treatment	*Inhibit BACE-1 activity	
Reverse scopolamine-induced increase in iron contents in the hippocampus	mouse	*25, 50 mg/kg	*Chelate intracellular Fe^2+^	Wang et al. (2017)
		*i.g	*Inhibit TrR1 expression	
		*Once daily, 6 days	—	

Mechanistic studies have shown that myricetin improves learning and memory impairment in rodent models of AD likely through reversing streptozotocin-induced decrease in neuronal numbers in the hippocampus (Ramezani et al., 2016) or D-galactose-induced decrease in the phosphorylation levels of ERK1/2 and CREB (Lei et al., 2012; Figure 3 and Table 2), two molecules that have neuroprotective functions. *In vitro* studies have reported that pre- or simultaneous administration of myricetin (0.3, 1, 3 and/or 10 μM, 48 h) can suppress Aβ_1-42_-induced nuclear fragmentation and caspase-3 activation in primary cultured cortical neurons, and reduce Aβ_1-40_/Aβ_1-42_ levels in neuronal culture media via the promotion of α-secretase (ADAM10) expression and the inhibition of β-secretase (BACE-1) activity (Shimmyo et al., 2008), suggesting that promoting neuronal survival and inhibiting Aβ accumulation may mediate the anti-AD’s effects of myricetin. In addition, myricetin administration has been shown to reverse D-galactose-induced decreases in SOD activity and increases in levels of thiobarbituric acid reactive substances (TBARS) (Lei et al., 2012; Figure 3 and Table 2), or scopolamine-induced increases in malondialdehyde (*MDA*) levels and decreases in SOD, glutathione peroxidase (GPx), and catalase activities in the hippocampus (Wang et al., 2017; Figure 3 and Table 2), suggesting that anti-oxidation may mediate the anti-AD’s effects of myricetin, at least in rodent models of D-galactose or scopolamine stimulation.

Iron over-accumulation has been shown to be actively involved in AD pathogenesis. Inhibition of iron accumulation may help to ameliorate AD symptoms (Li et al., 2017; Schröder et al., 2013). Myricetin treatment (25 or 50 mg/kg, once daily, 6 days) can reverse scopolamine-induced increases in iron contents in the hippocampus in mice via direct chelating of intracellular Fe^2+^ or inhibition of transferrin receptor 1 (TrR1) expression (Wang et al., 2017), suggesting that inhibition of iron accumulation may contribute to the neuroprotective effects of myricetin in AD. Cholinergic hypofunction is another pathological process in AD (Ahmed et al., 2017). In mouse models of AD, myricetin administration (25 or 50 mg/kg, once daily, 6 days) can prevent scopolamine-induced increases in acetylcholinesterase (AChE) activities, thereby inducing significant increases in acetylcholine contents in the hippocampus (Wang et al., 2017; Figure 3 and Table 2), suggesting that reversing cholinergic hypofunction may be a key mechanism for the anti-AD’s effects of myricetin. This hypothesis could be supported by studies *in vitro*: myricetin incubation (0.016, 0.063, 0.25, 1, and 4 μM) can inhibit the activity of AChE in a dose-dependent manner in SH-SY5Y cells (Wang et al., 2017).

## Pharmacological Effects of Myricetin in Parkinson’s Disease

**FIGURE 4:**
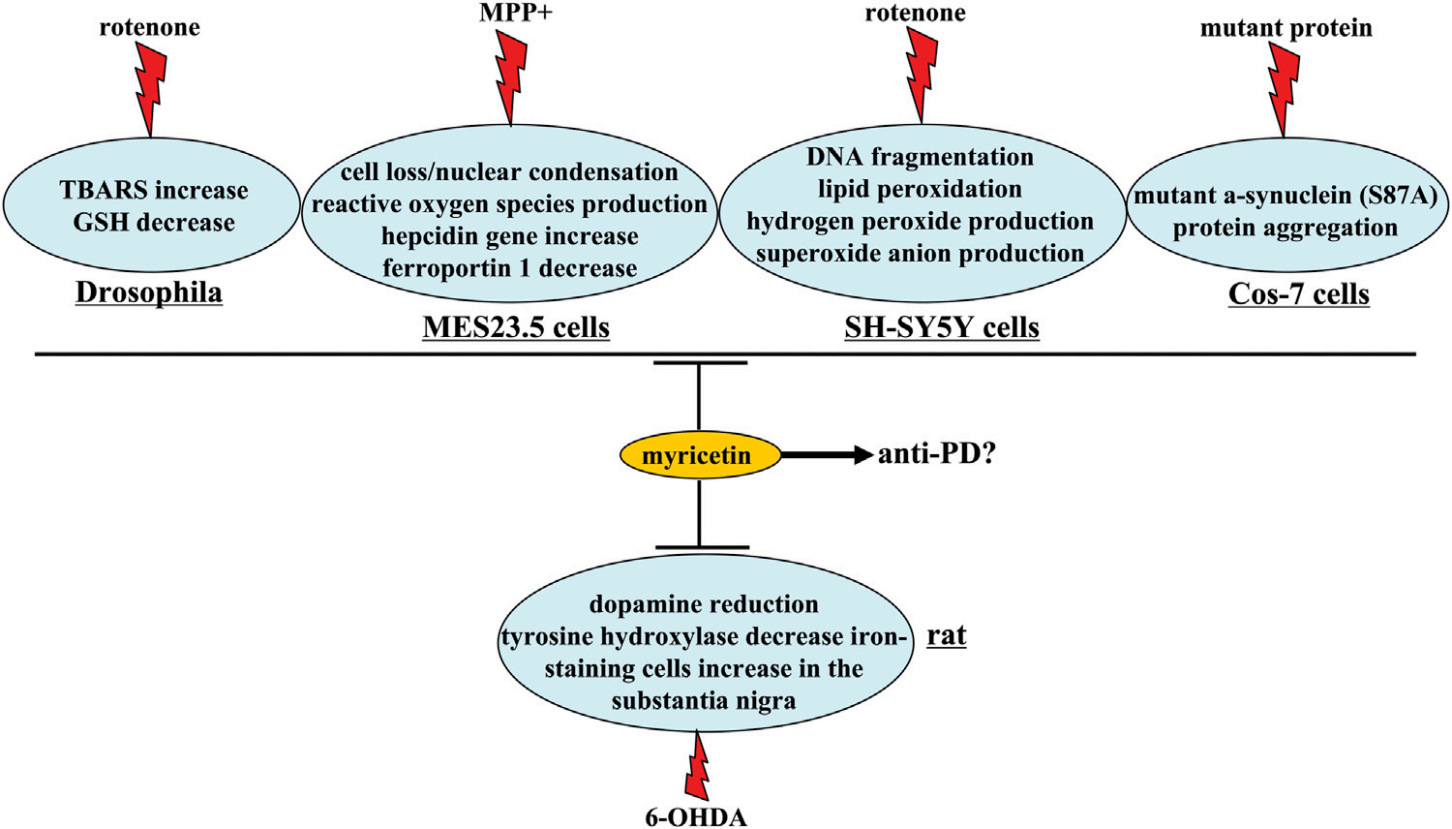
Neuroprotective effects and possible mechanisms of myricetin in Parkinson’s disease-associated models. Myricetin produces anti-PD’s effects via suppressing 6-OHDA-induced decrease in dopamine contents and tyrosine hydroxylase expression and increase in iron-staining cells in the substantia nigra. Myricetin also has protective effects in *Drosophila*, MES23.5 cells, SH-SY5Y cells, and Cos-7 cells, as indicated by the concrete descriptions in this figure. Whether these effects could mediate the anti-PD effect of myricetin remain unclear.

Parkinson’s disease (PD) is a neurodegenerative disease with dopaminergic neuronal degeneration in the Substantia Nigra pars compacta (SNc) and dopamine depletion in the striatum (Mishra et al., 2019; Serra et al., 2021). Besides the use of strategies that restore a normal dopamine signaling, no effective methods could be available for PD treatment in clinic. In a previous study, lateral cerebral ventricle injection of myricetin (0.5 mg/ml, 7 days) has been shown to reverse 6-hydroxydopamine (6-OHDA)-induced decreases in dopamine contents in the striatum in rats via increasing tyrosine hydroxylase expression in the substantia nigra (Ma et al., 2007; Figure 4 and Table 3). In a *Drosophila* model of PD, myricetin administration (314 mM, 3 h before rotenone exposure, 7 days) was found to suppress rotenone-induced gait disturbance, muscular dys-coordination, and memory impairment (Dhanraj et al., 2018; Figure 4 and Table 3).

**TABLE 3:** Comprehensive information about the effects of myricetin in animal models Parkinson’s disease.

Pharmacological effect	Object	Drug administration	Possible mechanisms	References
Reverse 6-OHDA-induced decrease in dopamine contents in the striatum	Rat	*0.5 mg/ml	Increase tyrosine hydroxylase expression and reduce iron-staining cells in the substantia nigra	Ma et al. (2007)
		*lateral cerebral ventricle injection		
		*7 days		
Suppress rotenone-induced gait disturbance, muscular dys-coordination, memory impairment, dopaminergic neuronal degeneration, and dopamine reduction	*Drosophila*	*314 mM	*Reduce TBARS levels and Bax expression	Dhanraj et al. (2018)
		*3 h before rotenone exposure	*Increase GSH levels and Bcl-2 expression	
		*7 days	—	
Attenuate MPP^+^-induced cell loss and nuclear condensation	MES23.5 cells	50 μM Co-treatment	*Restore mitochondrial transmembrane potential	Zhang et al. (2011)
			*Suppress reactive oxygen species production	
Prevent rotenone-induced cell loss	SH-SY5Y cells	*50 μM	*Suppress DNA fragmentation, lipid peroxidation, and the production of hydrogen peroxide and superoxide anion	Molina-Jiménez et al. (2004)
		*30 min before rotenone treatment		
Alleviate rotenone-induced decreases in cell viability	MES23.5 cells	*1 μM	*Suppress reactive oxygen species production	Deng et al. (2021)
		*24 h of pretreatment	*Restore mitochondrial transmembrane potential	
		—	*Reduce hepcidin gene transcription	
		—	*Increase ferroportin 1 expression	
Suppress the aggregation of the mutant α-synuclein (S87A) protein	Cos-7 cells	*10 μM	Activate the ubiquitin-proteasome pathway	Joshi et al. (2019)
		*48 h		

One of the mechanisms that mediate the anti-PD’s effects of myricetin may be associated with anti-oxidation. Firstly, in *Drosophila*, myricetin treatment (314 mM, 3 h before rotenone exposure, 7 days) was found to suppress rotenone-induced increases in TBARS levels and decreases in GSH levels (Dhanraj et al., 2018) (Figure 4). Secondly, myricetin co-incubation (50 μM) has been shown to attenuate 1-methyl-4-phenylpyridinium ion (MPP^+^)-induced cell loss and nuclear condensation by restoring mitochondrial transmembrane potential and suppressing the production of reactive oxygen species in MES23.5 cells, a type of cells with similar properties as the neurons in the substantia nigra (Zhang et al., 2011; Figure 4 and Table 3). Thirdly, myricetin incubation (50 μM, 30 min before rotenone treatment) in SH-SY5Y cells prevents rotenone-induced cell loss *via* suppressing DNA fragmentation, lipid peroxidation, and the production of hydrogen peroxide and superoxide anion (Molina-Jiménez et al., 2004; Figure 4 and Table 3).

Besides oxido-nitrosative stress and cellular apoptosis, iron accumulation is also known to mediate the pathogenesis of PD (Raj et al., 2021); suppression of iron accumulation may be a potential strategy for the treatment of PD. Myricetin treatment (lateral cerebral ventricle injection; 0.5 mg/ml, 7 days) can prevent 6-OHDA-induced increase of iron-staining cells in the substantia nigra of rats (Ma et al., 2007), and in MES23.5 *cells*, myricetin pre-treatment (1 μM, 24 h) has been reported to suppress rotenone-induced hepcidin gene transcription and decrease in ferroportin 1, an iron efflux transporter that can inhibit iron efflux (Deng et al., 2021; Figure 4 and Table 3), suggesting that inhibition of iron accumulation-associated pathologies in the substantia nigra may contribute to the anti-PD’s effects of myricetin. In addition, myricetin incubation (10 μM, 48 h) has been shown to suppress the aggregation of the mutant α-synuclein (S87A) protein in Cos-7 cells *via* the activation of the ubiquitin-proteasome pathway (Joshi et al., 2019; Figure 4 and Table 3). As the α-synuclein (S87A) mutant is tightly associated with PD (Oueslati et al., 2012), this finding demonstrated that inhibition of α-synuclein (S87A) aggregation may be another mechanism for the anti-PD’s effects of myricetin. In fact, inhibition of protein aggregation is a featured characteristic of myricetin, as myricetin incubation (10 μM, 48 h) can suppress the aggregation of the misfolded Δ9CAT mutant protein, thermally denatured misfolded protein, mutant SOD-1 protein associated with Amyotrophic Lateral Sclerosis, and expanded (HDQ74) polyglutamine protein associated with Huntington’s disease in Cos-7 cells (Joshi et al., 2019). Catechol O-methyltransferase (COMT) is an enzyme whose inhibition can increase the bioavailability of L-dopa (Bonifácio et al., 2007). Myricetin incubation (10, 20, or 50 μM) has been shown to inhibit the activity of COMT, suggesting that the previously-observed elevation of striatal dopamine levels in myricetin-treated rats is possibly mediated by the direct inhibition of myricetin on COMT activity (Zhu and Jia, 2014).

## Pharmacological Effects of Myricetin in Epilepsy

Epilepsy is a nervous system disorder characterized by the abnormal increase in neuronal excitability (Hamed, 2020). How to calm-down the over-activated neurons is always a research topic. Orally myricetin administration (100 or 200 mg/kg, 26 days, 30 min prior to each pentylenetetrazol injection) can reduce seizure rates in a mouse model of epilepsy induced by pentylenetetrazole likely through up-regulating the expression of Bcl-2, Bcl-xL, brain-derived neurotrophic factor (BDNF), glutamate decarboxylase 65 (GAD65), and γ-aminobutyric acid (GABA_A_), down-regulating the expression of caspase-3, Bad, and Bax, up-regulating the levels of GABA, and reducing glutamate contents and matrix metalloprotein-9 (MMP-9) activity in the hippocampus (Sun et al., 2019). Myricetin incubation (5 μg/ml) can also promote the GABAergic activity in hypothalamic paraventricular nucleus (PVN) neurons by increasing the decay time and frequency of the inhibitory currents mediated by the GABA_A_ receptor (Zhang et al., 2012). These effects of myricetin may be mediated by the Ca^2+^-Ca^2+^/calmodulin dependent protein kinase II (CaMKII) signaling, as myricetin can activate the T- and L-type Ca^2+^ channel and increase the levels of phospho-CaMKII (Zhang et al., 2012). Further analysis showed that the enhancing effect of myricetin on GABAergic activity was not mediated by antagonizing the GABA_A_ receptor benzodiazepine-binding site (Zhang et al., 2012), suggesting that the action mode of myricetin on the GABA_A_ receptor may be distinct from the most existing benzodiazepine-binding site agonists of GABA_A_ receptors. In addition, the same dose of myricetin incubation can enhance a type of potassium currents in hypothalamic type-I PVN neurons in a Ca^2+^-dependent manner, and this type of currents may be the Ca^2+^-activated potassium current, as these currents could be activated in a relative high voltage (more positive than −40 mV) and be inactivated slowly (Ma and Liu, 2012), which is in accordance with the property of the typical Ca^2+^-activated potassium current (Li and Ferguson, 1996).

## Pharmacological Effects of Myricetin in Glioma

Glioblastoma is the most common type of glioma with high proliferative activities. It can be treated by methods including chemotherapy, radiotherapy, and surgery (Cruz Da Silva et al., 2021). The current therapies are only partially useful and the median survival time of glioblastoma patients receiving the current therapies is lesser than 18 months (Otto-Meyer et al., 2020). Thus, it is urgent to search novel drugs for the treatment of glioblastoma. Myricetin may have a potential to be developed as a novel anti-tumor agent due to its remarkable anti-cancer properties. In a previous study, a sub-toxic dose of myricetin incubation (50 μM) has been shown to enhance tumor necrosis factor-related apoptosis-inducing ligand (TRAIL)-induced apoptosis of glioblastoma cells by augmenting the activation of caspases-3/-7/-8/-9 (Siegelin et al., 2009). Bcl-2 and the short isoform of c-FLIP, two proteins which can inhibit TRAIL-triggered cellular apoptosis, appear to mediate the sensitization effect of myricetin in glioblastoma cells, as their expression can be down-regulated by myricetin incubation (50 μM), and their overexpression can abrogate the facilitation effect of myricetin on TRAIL-induced apoptosis of glioblastoma cells (Siegelin et al., 2009). In another study by Zhao et al. (2018), myricetin incubation (≥20 µM) was found to inhibit glioblastoma cell proliferation, migration, and invasion by blocking the formation of lamellipodia, focal adhesions, membrane ruffles, and vasculogenic mimicry in a manner dependent on the suppression of Akt, c-Jun, Rho-associated protein kinase 2 (ROCK2), paxillin, and cortactin phosphorylation. In U251 human glioma cells, myricetin incubation (5, 15, 30, 60, 120, 240 μM) was found to alter the glioma cell shape, which is likely associated with the promotion of cellular apoptosis (Li et al., 2021). Medulloblastoma is a highly metastatic disease in children. The activation of the hepatocyte growth factor (HGF) signaling is a known mechanism for the progression of medulloblastoma; its inhibition is a potential strategy for the treatment of medulloblastoma (Onvani et al., 2012). Myricetin incubation (2.5, 5, 10, 20 μM) can prevent HGF-induced phosphorylation of Met, a tyrosine kinase receptor for HGF, in a medulloblastoma cell line (DAOY), and HGF-induced formation of membrane ruffles of DAOY cells, thereby suppressing the migration of DAOY cells and tumor invasion (Labbé et al., 2009).

## The Other Pharmacological Effects of Myricetin in the Nervous System

Multiple sclerosis is an autoimmune disease which can induce severe oxidative stress in the nervous system; rectifying the imbalance of oxidative stress may help to ameliorate the pathogenesis of multiple sclerosis (Tanaka and Vécsei, 2020). Orally myricetin administration (100 mg/kg, once daily, 5 weeks) has been shown to ameliorate hyper-locomotion, sustain the balance of mice for a longer time in a high-speed rotating cylinder, reverse spatial learning and memory damage, hinder myelin reduction, and promote myelination in cuprizone-treated mice by promoting the nuclear translocation of Nrf2 and heme oxygenase-1 (HO-1) and NAD(P)H:quinone oxidoreductase 1 (*NQ O 1*) expression, reducing MDA levels, and increasing SOD/catalase activities (Zhang et al., 2016). These results demonstrate that myricetin can ameliorate the neurological deficits in pathogenesis of multiple sclerosis via the Nrf-2-mediated anti-oxidative signaling, and myricetin could be developed as a novel drug for the treatment of multiple sclerosis.

Chronic stress exposure is a common pathological factor that can induce behavioral abnormalities indicating the occurrence of various psychological disorders in the modern society. Mechanisms that mediate stress-induced behavioral abnormalities are largely unknown. The dysfunctions of the endocrine system, oxidative stress, and neurotrophic factor can be used to explain the pathogenesis of stress-induced psychological disorders (Claudino et al., 2020; McEwen and Akil, 2020; Satoh, 2021). Myricetin administration (50 mg/kg, i. p., 21 days) has been shown to reduce repeated restraint stress-induced increases in immobility time in mice in the forced swimming and tail suspension test likely through reducing the plasma corticosterone levels and increasing hippocampal GSP-Px activity and BDNF expression in the hippocampus (Ma et al., 2015). Myricetin administration (40 min before each stress stimulation, 50 mg/kg, i. p., 21 days) can also inhibit repeated restraint stress-induced spatial memory in mice via reducing the levels of plasma adreno-corticotrophic hormone and increasing hippocampal BDNF expression, which was indicated by the increase in the time that spent in the target quadrant in mice exposed to chronic stress in the probe trial in the Morris water maze task (Wang et al., 2016).

Neuroinflammation is a common pathological change that mediates the progression of nervous system disorders (Meyer et al., 2020; Piancone et al., 2021). Inhibition of neuroinflammation is a potential strategy for the treatment of nervous system disorders. Myricetin incubation (50 or 100 μM) has been shown to skew the hypoxia-triggered neuroinflammatory response towards an anti-inflammatory M2 phenotype in BV-2 microglia, thereby protecting the SH-SY5Y cells from death induced by conditioned media from hypoxia-stimulated microglia (Boriero et al., 2021). Mechanistic studies have shown that myricetin incubation can reduce the phospho-signal transducer and activator of transcription 1 (STAT1) via a direct interaction with STAT1 protein (Boriero et al., 2021). This finding is line with a fact that STAT1 is a pivotal factor that regulates the transition of microglia towards a pro-inflammatory M1 phenotype under hypoxia in a phosphorylation-dependent manner (Butturini et al., 2019). Myricetin incubation (10 and 25 μM) can also down-regulate lipopolysaccharide (LPS)-induced increase in interleukin-1β (IL-1β), tumor necrosis factor-α (TNF-α), inducible nitric oxide synthase (iNOS), cyclooxygenase-2 (COX-2), and prostaglandin E2 (PGE2) levels in BV-2 microglia *via* inhibiting JNK and p38 phosphorylation (Jang et al., 2020), which could be evidenced by a direct inhibition of LPS-induced increase in microglial numbers in the hippocampus and prefrontal cortex (50 and 100 mg/kg, i. p., once daily, 7 days) (Jang et al., 2020).

## Conclusion

In this review, we outlined the pharmacological effects of myricetin in the nervous system. Myricetin can produce preventive and therapeutic effects in nervous system disorders through anti-oxidation (Wu et al., 2016), anti-proliferation (Siegelin et al., 2009; Zhao et al., 2018; Li et al., 2021), and anti-neuroinflammation (Jang et al., 2020; Boriero et al., 2021). The activation of the *Nrf2* (Wu et al., 2016), ERK1/2 (Wang et al., 2017; Jang et al., 2020), Akt (Sun et al., 2018), CREB (Lei et al., 2012), and BDNF (Sun et al., 2019), the inhibition of intracellular Ca^2+^ increase and JNK-p38 activation (Oyama et al., 1994; Hagenacker et al., 2010; Chang et al., 2015; Sun et al., 2018; Jang et al., 2020), and the suppression of mutant protein aggregation (Joshi et al., 2019) and PVN neuronal activity (Ma and Liu, 2012; Zhang et al., 2012) may also mediate the neuroprotective effects of myricetin in the nervous system. However, no exact causal relationship between myricetin and its neuroprotective effects has been established. If we want to develop myricetin as a novel drug for the treatment of nervous system disorders, we should clarify the common pathway that mediates the neuroprotective effects of myricetin. We should also notice the pharmacodynamic deficits of myricetin which may restrict its application in daily-life and clinical practice, and develop novel strategies to improve the oral bioavailability of myricetin and increase the stability of myricetin in gastric fluids. These endeavors appear to be very important, as although myricetin is not being applied for the treatment of nervous system disorders, some clinical studies have revealed promising potentials for its use in clinic. For example, Emulin™ which contains myricetin has been shown to reduce blood glucose increase in type 2 diabetic patients (Ahrens and Thompson, 2013). In a 4-weeks randomized placebo-controlled clinical trial, the Blueberin which contains 50 mg myricetin was found to reduce fasting plasma glucose and serum C-reactive proteins levels (Abidov et al., 2006). Some other studies had reported that myricetin intake is associated with lower cancer risk in humans (Knekt et al., 2002; Tang et al., 2009). Since inflammation, diabetes, and cancer are highly correlated with nervous system disorders, these previously-reported clinical studies provide indirect evidence for the development of clinical trials for myricetin in nervous system disorder treatment.
